# Association of Cancer with Heart Failure and the Prognostic Value of NT-proBNP in Cancer Patients: Findings from the NHANES (1999–2018)

**DOI:** 10.3390/curroncol31090365

**Published:** 2024-08-26

**Authors:** Qingping Zeng, Weihong Chang, Rui Zhang, Hongxuan Fan, Zixuan Dou, Aman Liu, Jie Yu, Boda Zhou

**Affiliations:** 1 School of Clinical Medicine, Tsinghua University, Beijing 102218, China; 2 Department of Cardiology, Beijing Tsinghua Changgung Hospital, School of Clinical Medicine, Tsinghua University, Beijing 102218, China; 3 School of Medicine, Tsinghua University, Beijing 100084, China; 4 Cardiovascular Department, John Hunter Hospital, Newcastle, NSW 2305, Australia; 5 The George Institute for Global Health, Faculty of Medicine, University of New South Wales, Sydney, NSW 2000, Australia

**Keywords:** heart failure, cancer, survival, NT-proBNP

## Abstract

Evidence regarding the association between cancer and heart failure (HF) is scarce. This study is to investigate the association between HF and cancer and explore the prognostic value of NT-proBNP in cancer patients. This cohort study used National Health and Nutrition Examination Survey data from 1999 to 2018 and linked mortality information until 2019. We included all participants with valid answer to questions regarding self-reported cancer and HF. Multivariable logistic regression was used to estimate odds ratios (ORs) and 95% CIs. Our study included data from 54,847 adult participants. During a median (IQR) follow-up of 9.6 (4.0–15.1) years, 7674 deaths were recorded. HF was associated with an increased occurrence of cancer after propensity score matching (OR = 1.46, 95% CI: 1.17–1.82, *p* < 0.001). Cancer was associated with a higher occurrence of HF (OR = 1.33, 95% CI: 1.11–1.59, *p* = 0.002). Kaplan–Meier survival analysis over 10 years revealed the shortest survival in patients with both HF and cancer (log-rank *p* < 0.0001). Importantly, NT-proBNP was significantly higher in cancer patients, no matter whether with known HF (*p* < 0.01). In cancer patients without HF, NT-proBNP higher than 51.51 pg/mL was associated with shorter survival (log-rank *p* < 0.0001). Findings from this cohort study suggest that HF is significantly associated with cancer. NT-proBNP was higher in cancer patients, with significant prognostic value in cancer patients.

## 1. Introduction

The number of newly diagnosed cancers has soared from 18.7 million in 2010 to 23.6 million in 2019, with an increase of 26.3%. At the same time, the global death from cancer rose from 8.29 million to 10 million, an increase of 20.9% in the last decade [1]. Compared to these cancer statistics, the global burden of HF ranges from 1–3% in the general adult population, from 1 to 20 cases per 1000 people, with a five-year mortality rate of 50 to 75% [2]. In clinical practice, we observed that cancer patients are prone to suffering from HF. In 2013, Tal Hasin and his team conducted the first in-depth study of the association between HF and cancer, showing that HF patients with tumors had an increased risk of death [3]. Recent research has shown that HF patients are more likely to develop cancer, but there is insufficient research evidence to prove whether there is an association between the two diseases and whether having both diseases results in an increase in mortality.

Cancer patients are prone to developing HF [4], which can be observed after a variety of treatments including chemotherapy, radiotherapy, targeted therapy and immunotherapy. Chemotherapy is widely considered an effective anti-cancer treatment. However, some medication, e.g., anthracycline, can cause damage to cardiomyocytes, leading to a decline in heart function and, ultimately, heart failure [4]. Radiotherapy is used to treat some types of cancer but can cause problems such as cardiomyopathy and pericarditis [5]. In addition, the heart can be adversely affected by radiation, which impairs heart function and increases the risk of heart failure. Certain targeted therapies can also have harmful effects on the heart, causing damage to the myocardium and reducing heart function. Inhibitors of vascular endothelial growth factor (VEGF), for example, may increase the risk of HF through a variety of mechanisms, including endothelial damage, vasoconstriction and remodeling, and inflammatory responses. Immunotherapy (for example, PD-1 inhibitors and CTLA-4 inhibitors) may also cause the immune system to attack heart tissue sometimes, which leads to myocarditis and compromised heart function. At the same time, cancer itself has the potential to affect heart function in a number of ways, such as cancerous cardiomyopathy and cancerous pericarditis.

HF may increase the risk of cancer, which may be attributed to factors such as weakened immune system and HF medication. HF patients are more likely to be attacked by tumor cells due to their dysfunctional immune system, leading to increased incidence of cancer [6]. Some HF medications are thought to be associated with the development and progression of cancer, especially in patients who take certain medications for long periods of time. In a national insurance cohort study in South Korea, which investigated cancer risk in HF patients, found that valsartan, which is used in HF patients, increased the risk of liver and kidney cancer compared to other ARBs [7]. In addition, there are many common risk factors between cardiovascular disease and cancer, such as age, smoking, overweight, diabetes, and hyperlipidemia [8]. However, a clear association between the two diseases has yet to be established, and markers for early screening of HF in cancer patients have not been determined [8]. Therefore, the purpose of this study was to determine whether there is an association between HF and cancer, as well as its impact on survival, and to further explore whether NT-proBNP level in cancer patients are predictive of HF in NHANES database between 1999 and 2018.

## 2. Methods

### 2.1. Study Population

As noted elsewhere, the National Health and Nutrition Examination Survey (NHANES) is a nationwide, population-based, multiyear cross-sectional study. The study sample is a representative sample of the U.S. civilian population that is not institutionalized. Demographic information (for more information, visit http://wwwn.cdc.gov/nchs/nhanes/, accessed on 1 August 2024), as well as self-reported health status with or without cancer (MCQ220) and heart failure (MCQ160B), were obtained through home interviews using questionnaires whose data were well preserved and shipped to relevant health research centers for analysis. Detailed data processing and analysis methods can be found on the NHANES website (for more information, visit https://wwwn.cdc.gov/nchs/nhanes/2017-2018/MCQ_J.htm, accessed on 1 August 2024). For the current analysis, a 1999–2018 cohort was selected to study the relationship between cancer and heart failure. NHANES researchers received informed consent from participants.

### 2.2. Data Collection

The present study collected data from participants between the age of 20 and 85. Before statistical analysis, we excluded participants who had missing data in either MCQ220 or MCQ160B from the analysis. For data missing from variables other than MCQ220 and MCQ160B, we treated the missing data separately depending on whether the variable was categorical or continuous. For categorical variables, individuals with missing data were categorized as a separate group. For continuous variables, we deleted the variable when the missing rate was >40%; we used mean values to fill missing data when the missing rate was less than 5%; and we used multiple insertions to fill missing data when the missing rate was between 5% and 40%.

### 2.3. NT-proBNP Measurement

We used serum NT-proBNP data from the 1999–2004 NHANES cycle with a range of 5 pg/mL to 35,000 pg/mL. In addition, we explored significant differences in NT-proBNP levels between different HF and cancer groups using ANOVA and Tukey multiplex comparison assays.

### 2.4. Statistical Analysis

In this study, we first performed a detailed descriptive statistical analysis of baseline characteristics of included participants. These characteristics include important demographic variables such as age, gender, race, and education. For categorical variables, we displayed percentage (95% CI). For continuous variables following a normal distribution, we calculated the mean and standard deviation (mean ± SD). However, due to the significant skewness of NT-proBNP in the NHANES data, we used the mean and standard error of the mean (mean ± SEM) for its representation.

### 2.5. Preference Score Matching (PSM)

To further analyze the association between heart failure and cancer, we performed a bias score matching (PSM) application. PSM is a statistical method used to reduce the impact of potentially confounding variables in observational studies by matching participants from different groups (e.g., with or without heart failure, cancer, etc.) to ensure similarity in key covariates. Before and after PSM, we performed a balancing test to ensure the validity and accuracy of PSM. The specific operations of the match include the use of the nearest neighbor method and standardized calibration value settings, as well as careful balancing assessment of the match results. Comparing baseline features, we evaluated significant differences between groups using the Kruskal–Wallis rank sum test and/or Fisher precision probability test.

### 2.6. Logical Regression Model

To comprehensively assess the association between self-reported cancer and heart failure in adults, we used a survey-weighted logistic regression model based on NHANES data. For our regression analysis, we used weighted variables designed by NHANES; considering our study included hematology variables, we chose mobile exploration center (MEC) weights. The weight calculation formula for 1999–2000 and 2001–2002 was 2/10 × wtmec4yr, and the weight calculation formula for 2003–2018 was 1/10 × wtmec2yr. We constructed multiple models to explore the relationship between heart failure and cancer. For example, we present three weighted logistic regression models before and after PSM. Model 1 analyzed the coefficient of cancer on HF; model 2 added covariates such as gender, age, race and education to model 1. Model 3 further added variables such as diabetes, systolic blood pressure, diastolic blood pressure, and BMI to model 2. Overall, the results of these weighted logistic regression models provide odds ratio (OR) and 95% confidence interval (CI) and P values for each variable, further enhancing the interpretation of the results. In this way, we were able to effectively assess and quantify the association between cancer and heart failure, taking into account multiple possible confounding factors.

### 2.7. Kaplan–Meier Curve Survival Analysis

Survival analysis was conducted using the Kaplan–Meier method to estimate survival probabilities for each group. Differences between groups were assessed with the log-rank test, with corresponding *p*-values reported. The KM curves are presented with 95% confidence intervals.

We use R software (version 4.3.1) and Empower 6.0 for all statistical analysis.

## 3. Results

### 3.1. Baseline Characteristics

**Figure 1:**
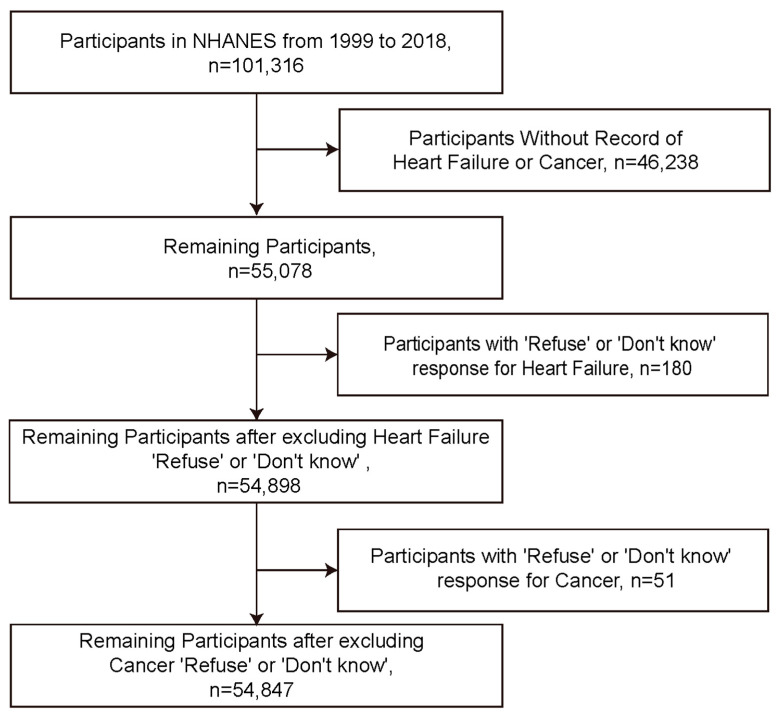
Flowchart of participant inclusion.

This study finally included 54,847 participants in the NHANES cohort from 1999 to 2018 (Figure 1). We first compared weighted baseline characteristics based on the presence or absence of HF (Table 1). Before propensity score matching (PSM), marked differences were observed between HF and non-heart failure (non-HF) groups in terms of age (*p* < 0.0001), poverty income ratio (*p* < 0.0001), body mass index (BMI) (*p* < 0.0001), cancer (*p* < 0.0001), gender (*p* = 0.0065), race (*p* < 0.0001), education years (*p* < 0.0001), marital status (*p* < 0.0001), diabetes (*p* < 0.0001), smoking (*p* < 0.0001), exercise habits (*p* < 0.0001), hepatitis C virus status (*p* = 0.004), and hepatitis B virus status (*p* = 0.8222). After PSM, disparities were resolved between the HF and non-HF groups. The majority of baseline characteristics between the HF and non-HF groups no longer showed significant differences, including age (*p* = 0.107), poverty income ratio (*p* = 0.734), gender (*p* = 0.624), race (*p* = 0.898), education years (*p* = 0.743), marital status (*p* = 0.684), diabetes (*p* = 0.556), smoking habits (*p* = 0.966), exercise habits (*p* = 0.066), hepatitis C virus (*p* = 0.361), and hepatitis B virus (*p* = 0.289). We also compared weighted baseline characteristics based on the presence or absence of cancer before and after PSM (Table 2).

### 3.2. Association between HF and Cancer

First, we constructed 3 logical regression models using HF as an outcome variable and cancer as a covariate before and after PSM (Table 3). In the pre-PSM analysis, Model 1 (unadjusted) demonstrated that cancer was significantly associated with HF (OR = 3.23, 95% CI = 2.77–3.77, *p* < 0.001). Model 2 adjusted for additional variables including gender, age, race, education, poverty income ratio, and marital status (OR = 1.41, 95% CI = 1.18–1.67, *p* < 0.001). Model 3 made further adjustments, including diabetes, blood pressure, smoking, body mass index, and waist circumference, on the basis of Model 2, which showed significant association between cancer and HF (OR = 1.33, 95% CI = 1.11–1.59, *p* = 0.002). After PSM, Models 1, 2, and 3 found that the association between cancer and HF was not statistically significant using HF as outcome and cancer as covariate.

Second, we constructed 3 logical regression models using cancer as an outcome and HF as a covariate before and after PSM (Table 4). In the pre-PSM analysis, Model 1 (unadjusted) demonstrated that HF was significantly associated with cancer (OR = 3.23, 95% CI = 2.77–3.77, *p* < 0.001). Model 2 adjusted for additional variables including gender, age, race, education, poverty income ratio, and marital status (OR = 1.39, 95% CI = 1.18–1.64, *p* < 0.001). Model 3 made further adjustments including diabetes, smoking, moderate activity, hepatitis C RNA, and hepatitis B surface antigen on the basis of Model 2, which showed significant association between HF and cancer (OR = 1.31, 95% CI = 1.11–1.55, *p* = 0.002). Following PSM, Model 3, with all the additional adjustments from the pre-PSM Model 3, showed significant association between HF and cancer (OR = 1.46, 95% CI = 1.17–1.82, *p* < 0.001).

### 3.3. Prevalence of HF and Diagnostic Value of NT-proBNP in Cancer Patients

**Figure 2:**
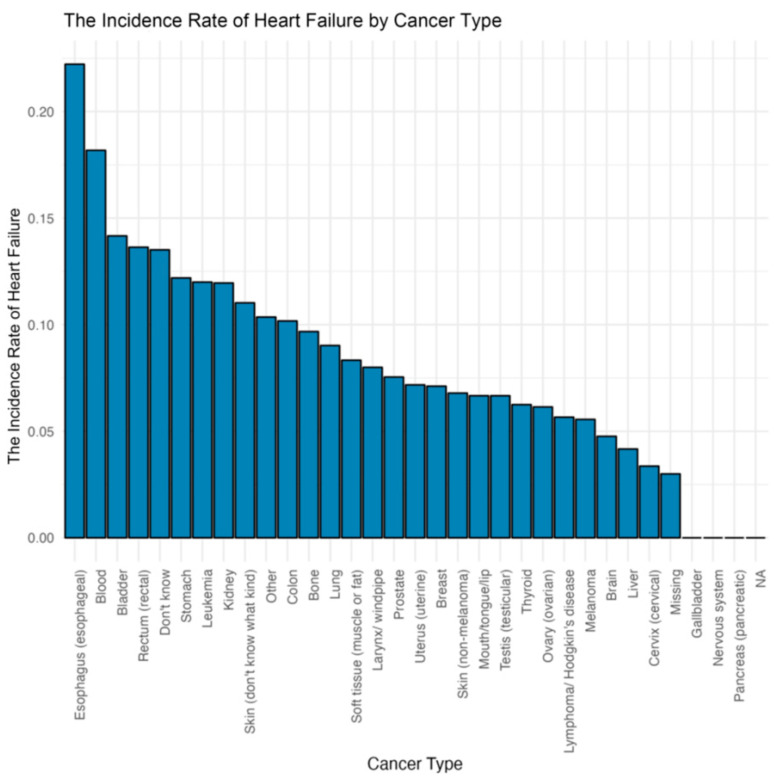
Incidence rate of heart failure among all participants with/without cancer.

**Figure 3:**
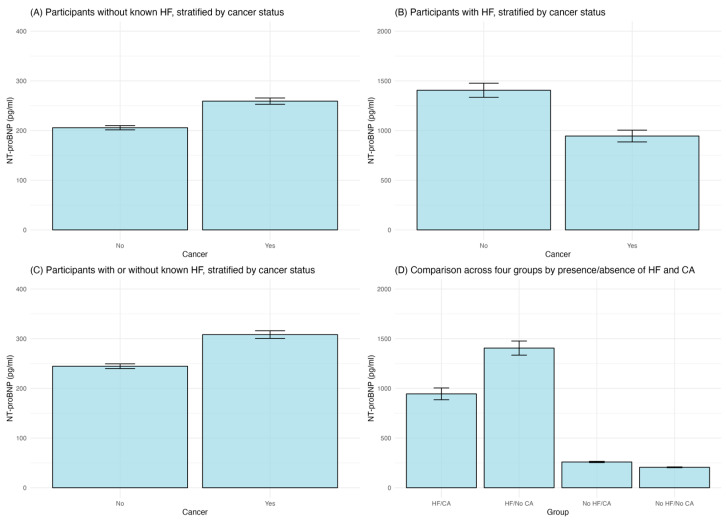
Comparison of NT-proBNP levels. (**A**). in participants without known HF, stratified by cancer status; (**B**). in participants with HF, stratified by cancer status; (**C**). in participants no matter whether they have known HF or not, stratified by cancer status; (**D**). across four patient groups categorized by the presence or absence of HF and cancer (CA).

Different cancer types were associated with different HF prevalence; esophagus and blood cancers were associated with higher HF prevalence (Figure 2). As shown in Figure 3, in participants without known HF, those with a cancer diagnosis exhibited significantly higher (*p* < 0.01) average levels of NT-proBNP (316.33 pg/mL) than those without cancer (151.90 pg/mL). In patients with known HF, those with concurrent cancer demonstrated significantly (*p* < 0.01) elevated NT-proBNP levels, with an average of 1597.50 pg/mL compared to 1114.89 pg/mL without cancer. Patients with cancer (412.36 pg/mL) had a significantly higher mean NT-proBNP level of compared to those without cancer (178.40 pg/mL), no matter whether with known HF (*p* < 0.01). Participants with both HF and cancer (HF/CA) displayed the highest average NT-proBNP level, while the group with neither condition (No HF/No CA) had the lowest average NT-proBNP level. The ANOVA results revealed significant differences between the four groups (F = 145.5, *p* < 0.01), while Tukey’s post hoc tests confirmed significant disparities between each pair of groups (all *p* < 0.01).

### 3.4. Prognostic Value of NT-proBNP in Cancer Patients

**Figure 4:**
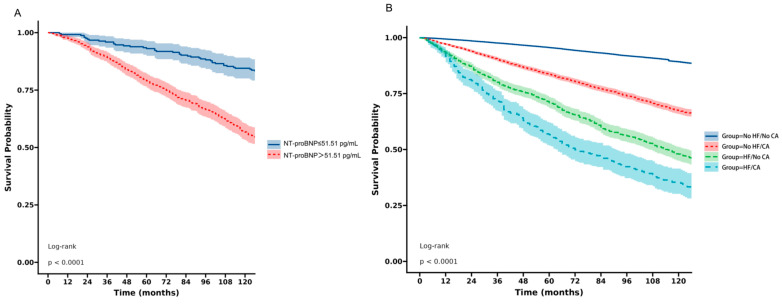
Kaplan–Meier survival analysis. (**A**) in cancer participants without known HF according to NT-proBNP level; (**B**) across four patient groups categorized by the presence or absence of HF and cancer (CA).

In order to explore the prognostic value of NT-proBNP in cancer patients, Kaplan–Meier survival analysis was performed in cancer participants without known HF (Figure 4). Patients were divided into two groups according to median NT-proBNP level: those with NT-proBNP≤ 51.51 pg/mL (low NT-proBNP group) and those with NT-proBNP > 51.51 pg/mL (high NT-proBNP group). The high NT-proBNP group showed significantly poorer survival compared with the low NT-proBNP group. The log-rank test confirmed a significant difference (*p* < 0.0001). We further categorized patients into four distinct groups based on the presence or absence of heart failure (HF) and cancer (CA). The Kaplan–Meier survival analysis compared survival between the four groups over the course of 120 months. Participants with no HF and no cancer (No HF/No CA) exhibited the highest survival. Conversely, participants with both HF and cancer (HF/CA) exhibited the lowest survival; a log-rank test showed significant difference among the four groups (*p* < 0.0001) (Figure 4).

## 4. Discussion

The aim of this study was to comprehensively assess the association between cancer and HF, with consequent risk of death in U.S. population, further explore the prognostic value of NT-proBNP in cancer patients. This study followed a rigorous research program and quality control process, with a large representative sample of data, which successfully captured multiple key covariates through the integration of NHANES data. As a result, many known confounding factors for cancer and HF were adjusted for in our analysis. We also used PSM to further control important possible confounding factors.

Based on the presence or absence of HF and cancer, we employed weighted logistic regression models to analyze the association between cancer and HF. The results showed significant association using cancer as outcome and HF as covariate after PSM and adjusting for known risk factors of HF/cancer, while no significant association persisted using HF as outcome and cancer as covariate after PSM and adjusting for known risk factors of HF/cancer. Our results confirmed a significant association between HF and cancer in a representative population. Surprisingly, through a two-way interaction analysis, this association may be attributed to the occurrence of cancer in HF patients.

Our in-depth study of the association between different types of cancer and HF found that esophageal and blood cancers were associated with higher risk of heart failure compared with other types of cancer. Moreover, this study found the longest median survival time in participants without HF or cancer and the shortest median survival time in participants with HF and cancer. Interestingly, participants with HF but without cancer showed significantly longer median survival time than participants with cancer but without HF. The poor prognosis in participants with HF and cancer may be attributed to several factors. Cancer patients with combined HF may not be able to tolerate invasive treatments such as surgery due to factors such as reduced heart function, which may influence the treatment effect of cancer and lead to poor prognosis. Moreover, certain cancer treatments, such as chemotherapy, immunotherapy or radiotherapy, may aggravate or cause HF and result in adverse outcomes [9,10].

In order to distinguish cancer patients with poor prognosis, we compared NT-proBNP levels in different groups. This study found significantly higher levels of NT-proBNP in participants with cancer than those without cancer, no matter whether they were known to have HF or not. Cancer participants without known HF and whose NT-proBNP level was higher than 51.51 pg/mL exhibited significantly shorter survival time. These results suggest that NT-proBNP could act as a prognostic marker in cancer patients. However, the cut-off value of NT-proBNP needs to be systematically evaluated in the cancer population for prognosis, with or without HF, and its predictive value for the development of ventricular dysfunction, even asymptomatic, needs further investigation.

Previous studies have shown that the incidence of cardiovascular disease in cancer patients is almost twice as high as in the general population, providing evidence of higher cardiovascular mortality in cancer patients [11,12]. A study showed that patients with uterine cancer who had undergone radiotherapy were more likely to die 10 years later from intercurrent conditions, including heart failure, cardiac arrhythmia, cardiac arrest and other complications, than from cancer [13]. Several studies have explored the prognostic value of NT-proBNP in cancer patients. A study directed towards cardiotoxicity monitoring found that NT-proBNP levels predicted cardiotoxicity in patients receiving anthracycline-based chemotherapy [14]. Another study was addressed to the utility of NT-proBNP in predicting adverse outcomes in cancer patients undergoing treatment, indicating its potential role in guiding therapy adjustments [15]. Finally, other studies showed that in patients with hematologic malignancies, the changes in NT-proBNP correlated with treatment efficacy and cardiac function [16,17]. However, so far no large-scale studies have demonstrated the bidirectional relationship between cancer and heart failure and the predictive role of NT-proBNP in cancer populations. Our study bridges this gap.

Taken together, the current study revealed significant association between cancer and HF in a representative large population cohort, co-morbidity of cancer and HF leads to an increased risk of death. Significantly higher level of NT-proBNP was found in participants with cancer than those without cancer, no matter they have known HF or not. In cancer participants without known HF higher NT-proBNP levels may indicate poor prognosis. This study supports the literature on the relationship between cancer and HF, which highlights the importance of early diagnosis and treatment of HF in cancer patients, as well as the need to emphasize cancer risk in patients with HF.

## 5. Limitations

Firstly, due to limitations of current cross-sectional data, we cannot draw definitive cause-and-effect conclusions. Secondly, as HF is identified by self-report, a definitive diagnosis of HF is limited. Thirdly, the NHANES database does not collect echocardiographic data and definitive etiologies of heart failure. Thus, the study did not specify the etiologies of HF (such as hypertension, coronary artery disease, valvular disease, cardiomyopathy, or atrial fibrillation) and type of HF (such as heart failure with reduced ejection fraction or heart failure with preserved ejection fraction). Fourthly, the observational design of this study could not exclude the uncontrolled latent biases when conducting a PSM.

## 6. Conclusions

Our study revealed that HF was significantly associated with cancer. NT-proBNP was higher in cancer patients, with significant prognostic value in cancer patients.

## Figures and Tables

**Table 1:** Baseline characteristics of participants based on heart failure status.

	Before Propensity Score Matching			After Propensity Score Matching		
	Non-HF (52,949)	HF (1898)	*p*-Value	Non-HF (1898)	HF (1898)	*p*-Value
Age (years)	46.5 (46.2, 46.9)	66.4 (65.6, 67.3)	<0.0001	69.0 (12.7)	68.4 (12.5)	0.107
Poverty income ratio	3.0 (2.9, 3.0)	2.3 (2.2, 2.4)	<0.0001	2.1 (1.3)	2.1 (1.3)	0.734
Body Mass Index (kg/m^2^)	28.7 (28.6, 28.8)	31.5 (31.0, 32.1)	<0.0001	29.3 (6.5)	31.2 (8.0)	<0.001
Cancer			<0.0001			0.029
No	90.8 (90.5, 91.2)	75.4 (72.6, 78.0)		1545 (81.4%)	1491 (78.6%)	
Yes	9.2 (8.8, 9.5)	24.6 (22.0, 27.4)		353 (18.6%)	407 (21.4%)	
Gender			0.0065			0.624
Female	52.1 (51.6, 52.5)	48.1 (45.3, 50.9)		842 (44.4%)	857 (45.2%)	
Male	47.9 (47.5, 48.4)	51.9 (49.1, 54.7)		1056 (55.6%)	1041 (54.8%)	
Race			<0.0001			0.898
Mexican American	8.3 (7.3, 9.4)	4.1 (3.1, 5.4)		219 (11.5%)	206 (10.9%)	
Other Hispanic	5.7 (4.9, 6.6)	4.3 (2.9, 6.3)		112 (5.9%)	117 (6.2%)	
Non-Hispanic White	67.9 (65.8, 69.9)	72.2 (69.2, 75.0)		1021 (53.8%)	1022 (53.8%)	
Non-Hispanic Black	11.2 (10.1, 12.4)	14.5 (12.4, 16.8)		442 (23.3%)	457 (24.1%)	
Other Race—Including Multi-Racial	7.0 (6.4, 7.7)	4.9 (3.6, 6.7)		104 (5.5%)	96 (5.1%)	
Education (years)			<0.0001			0.743
Less Than 9th Grade	5.9 (5.5, 6.3)	14.1 (12.4, 16.1)		408 (21.5%)	390 (20.5%)	
9–11th Grade (includes 12th grade with no diploma)	11.3 (10.7, 11.9)	18.3 (16.0, 20.9)		383 (20.2%)	385 (20.3%)	
High School Grad/GED or Equivalent	24.0 (23.2, 24.8)	27.2 (24.7, 29.9)		430 (22.7%)	460 (24.2%)	
Some College or AA degree	30.8 (30.1, 31.5)	27.5 (25.0, 30.1)		460 (24.2%)	461 (24.3%)	
College Graduate or Above	28.0 (26.7, 29.5)	12.8 (10.5, 15.6)		217 (11.4%)	202 (10.6%)	
Marriage			<0.0001			0.684
Married	56.1 (55.1, 57.1)	50.8 (47.4, 54.3)		911 (48.0%)	882 (46.5%)	
Widowed	5.8 (5.5, 6.0)	23.6 (21.1, 26.3)		514 (27.1%)	500 (26.3%)	
Divorced	9.9 (9.5, 10.3)	12.7 (11.0, 14.7)		225 (11.9%)	255 (13.4%)	
Separated	2.5 (2.3, 2.7)	2.8 (2.0, 3.8)		63 (3.3%)	66 (3.5%)	
Never Married	18.1 (17.3, 19.0)	6.9 (5.5, 8.7)		136 (7.2%)	139 (7.3%)	
Living with partner	7.6 (7.2, 8.1)	3.1 (2.2, 4.5)		49 (2.6%)	56 (3.0%)	
Diabetes			<0.0001			0.556
No	90.0 (89.6, 90.4)	59.9 (57.2, 62.7)		1075 (56.6%)	1087 (57.3%)	
Yes	8.2 (7.8, 8.5)	37.3 (34.5, 40.2)		752 (39.6%)	752 (39.6%)	
Borderline	1.8 (1.6, 2.0)	2.7 (2.0, 3.7)		71 (3.7%)	59 (3.1%)	
Missing	0.0 (0.0, 0.1)	0.0 (0.0, 0.0)				0.966
Smoking			<0.0001	790 (41.6%)	798 (42.0%)	
Not at All	24.2 (23.5, 24.9)	42.0 (38.8, 45.4)		53 (2.8%)	51 (2.7%)	
Some Days or	3.8 (3.5, 4.0)	2.8 (1.9, 4.1)		286 (15.1%)	276 (14.5%)	
Every Day	17.8 (17.1, 18.5)	16.2 (14.1, 18.5)		769 (40.5%)	773 (40.7%)	
Missing	54.3 (53.3, 55.2)	39.0 (36.0, 42.1)				0.066
Exercise			<0.0001	937 (49.4%)	896 (47.2%)	
No	42.8 (42.0, 43.6)	46.6 (43.6, 49.6)		561 (29.6%)	542 (28.6%)	
Yes	47.9 (47.0, 48.9)	29.2 (26.7, 31.9)		400 (21.1%)	460 (24.2%)	
Missing	9.2 (8.7, 9.8)	24.2 (21.3, 27.3)				
Hepatitis C Virus			0.004			0.361
Negative	0.8 (0.7, 0.9)	1.3 (0.8, 2.0)		25 (1.3%)	25 (1.3%)	
Positive	0.9 (0.8, 1.1)	1.7 (1.0, 2.9)		42 (2.2%)	30 (1.6%)	
Missing	98.3 (98.2, 98.5)	97.0 (95.7, 97.9)		1831 (96.5%)	1843 (97.1%)	
Hepatitis B Virus			0.8222			0.289
Negative	75.0 (73.7, 76.2)	74.6 (71.2, 77.8)		1319 (69.5%)	1274 (67.1%)	
Positive	0.3 (0.3, 0.4)	0.4 (0.1, 1.5)		6 (0.3%)	7 (0.4%)	
Missing	24.7 (23.4, 26.0)	24.9 (21.8, 28.3)		573 (30.2%)	617 (32.5%)	

The study compared the baseline characteristics of patients with heart failure (HF, *n* = 1898) to those without (Non-HF, *n* = 52,949) using propensity score matching. Before matching, continuous variables were assessed using weighted linear regression, while categorical ones were evaluated through weighted chi-square tests. The analysis provided weighted means and their 95% confidence intervals (CI) for continuous variables, and weighted percentages with 95% CI for categorical variables. However, post-matching, effective analysis of weighted baseline characteristics was hindered due to certain strata containing only one primary sampling unit (PSU). Hence, continuous variables were represented by mean(SD), and categorical variables by count and percentage (N(%)). For continuous variables, the Kruskal–Wallis rank-sum test was used to calculate *p*-values. For count variables with theoretical counts less than 10, Fisher’s exact test determined *p*-values.

**Table 2:** Baseline characteristics of participants based on cancer status.

	Before Propensity Score Matching			After Propensity Score Matching		
	Non-Cancer (49,709)	Cancer (5138)	*p*-Value	Non-Cancer (5138)	Cancer (5138)	*p*-Value
Heart Failure			<0.0001			0.2232
No	98.0 (97.8, 98.1)	93.8 (92.9, 94.6)		94.4 (93.6, 95.1)	93.8 (92.9, 94.6)	
Yes	2.0 (1.9, 2.2)	6.2 (5.4, 7.1)		5.6 (4.9, 6.4)	6.2 (5.4, 7.1)	
Age (years)	45.4 (45.0, 45.7)	62.5 (61.9, 63.1)	<0.0001	62.0 (61.3, 62.8)	62.5 (61.9, 63.1)	0.3011
Poverty Income Ratio	2.9 (2.9, 3.0)	3.2 (3.1, 3.2)	<0.0001	3.2 (3.1, 3.2)	3.2 (3.1, 3.2)	0.962
Systolic: Blood Pressure (first reading) mm Hg	122.1 (121.8, 122.4)	128.4 (127.6, 129.1)	<0.0001	128.1 (127.3, 128.9)	128.4 (127.6, 129.1)	0.5784
Diastolic: Blood Pressure (first reading) mm Hg	71.1 (70.8, 71.4)	69.1 (68.6, 69.6)	<0.0001	69.0 (68.4, 69.6)	69.1 (68.6, 69.6)	0.7533
Body Mass Index (kg/m^2^)	28.8 (28.6, 28.9)	28.8 (28.5, 29.0)	0.9909	28.9 (28.7, 29.2)	28.8 (28.5, 29.0)	0.3482
Waist Circumference (cm)	98.2 (97.8, 98.5)	101.0 (100.4, 101.5)	<0.0001	101.1 (100.5, 101.8)	101.0 (100.4, 101.5)	0.7202
Gender			<0.0001			0.7395
Female	51.4 (51.0, 51.8)	57.5 (55.8, 59.2)		57.1 (55.3, 58.8)	57.5 (55.8, 59.2)	
Male	48.6 (48.2, 49.0)	42.5 (40.8, 44.2)		42.9 (41.2, 44.7)	42.5 (40.8, 44.2)	
Race			<0.0001			0.6624
Mexican American	8.8 (7.7, 10.0)	2.3 (1.9, 2.9)		2.1 (1.7, 2.6)	2.3 (1.9, 2.9)	
Other Hispanic	6.0 (5.1, 7.0)	2.4 (1.8, 3.3)		2.5 (2.0, 3.1)	2.4 (1.8, 3.3)	
Non-Hispanic White	66.0 (63.9, 68.1)	86.3 (84.7, 87.7)		86.3 (84.8, 87.7)	86.3 (84.7, 87.7)	
Non-Hispanic Black	11.9 (10.8, 13.1)	5.6 (4.8, 6.4)		6.0 (5.2, 7.0)	5.6 (4.8, 6.4)	
Other Race-Including Multi-Racial	7.3 (6.7, 8.0)	3.4 (2.7, 4.3)		3.1 (2.5, 3.7)	3.4 (2.7, 4.3)	
Education (years)			<0.0001			0.9979
Less Than 9th Grade	6.2 (5.7, 6.6)	5.3 (4.7, 6.0)		5.2 (4.5, 5.9)	5.3 (4.7, 6.0)	
9–11th Grade (Includes 12th grade with no diploma)	11.6 (11.0, 12.3)	9.6 (8.5, 10.8)		9.5 (8.5, 10.6)	9.6 (8.5, 10.8)	
High School Grad/GED or Equivalent	24.2 (23.4, 25.0)	22.5 (20.8, 24.2)		22.5 (20.9, 24.1)	22.5 (20.8, 24.2)	
Some College or AA degree	30.7 (30.0, 31.5)	30.8 (29.0, 32.6)		31.1 (29.0, 33.2)	30.8 (29.0, 32.6)	
College Graduate or Above	27.2 (25.9, 28.6)	31.8 (29.4, 34.3)		31.7 (29.6, 34.0)	31.8 (29.4, 34.3)	
Marriage			<0.0001			0.3397
Married	55.3 (54.2, 56.3)	62.4 (60.5, 64.3)		61.0 (59.1, 62.9)	62.4 (60.5, 64.3)	
Widowed	5.3 (5.0, 5.6)	14.4 (13.3, 15.6)		13.9 (12.7, 15.1)	14.4 (13.3, 15.6)	
Divorced	9.8 (9.4, 10.2)	11.9 (10.8, 13.2)		13.1 (11.8, 14.6)	11.9 (10.8, 13.2)	
Separated	2.6 (2.4, 2.8)	2.1 (1.6, 2.6)		2.7 (2.1, 3.3)	2.1 (1.6, 2.6)	
Never Married	19.1 (18.2, 20.0)	5.7 (4.8, 6.6)		5.4 (4.6, 6.3)	5.7 (4.8, 6.6)	
Living with Partner	7.9 (7.5, 8.4)	3.5 (2.9, 4.1)		3.9 (3.2, 4.8)	3.5 (2.9, 4.1)	
Diabetes			<0.0001			0.6562
No	90.1 (89.7, 90.5)	81.5 (80.2, 82.8)		81.4 (80.0, 82.8)	81.5 (80.2, 82.8)	
Yes	8.2 (7.8, 8.5)	15.2 (14.1, 16.4)		15.7 (14.4, 17.1)	15.2 (14.1, 16.4)	
Borderline	1.7 (1.5, 1.8)	3.2 (2.6, 4.0)		2.8 (2.2, 3.4)	3.2 (2.6, 4.0)	
Missing	0.0 (0.0, 0.1)	0.0 (0.0, 0.2)		0.1 (0.0, 0.4)	0.0 (0.0, 0.2)	
Smoking			<0.0001			0.8497
Not at all	23.2 (22.5, 23.8)	38.4 (36.6, 40.2)		38.5 (36.6, 40.5)	38.4 (36.6, 40.2)	
Some Days or	3.9 (3.7, 4.1)	2.2 (1.7, 2.8)		2.3 (1.8, 2.9)	2.2 (1.7, 2.8)	
Every Day	18.1 (17.4, 18.9)	14.4 (13.0, 15.9)		13.6 (12.4, 14.9)	14.4 (13.0, 15.9)	
Missing	54.8 (53.9, 55.8)	45.0 (43.2, 46.9)		45.6 (43.6, 47.6)	45.0 (43.2, 46.9)	

This study utilized propensity score matching to compare baseline characteristics between patients with cancer (*n* = 5138) and those without (*n* = 49,709). Continuous variables were analyzed using survey-weighted linear regression, while categorical variables were evaluated by survey-weighted chi-square tests. The analysis provided survey-weighted means with 95% confidence intervals (CIs) for continuous variables and survey-weighted percentages with 95% CIs for categorical variables.

**Table 3:** Weighted logistic regression results for cancer status on HF.

	Model 1 Pre-PSM			Model 2 Pre-PSM			Model 3 Pre-PSM			Model 1 Post-PSM			Model 2 Post-PSM			Model 3 Post-PSM		
Characteristic	OR	95% CI	*p*-Value	OR	95% CI	*p*-Value	OR	95% CI	*p*-Value	OR	95% CI	*p*-Value	OR	95% CI	*p*-Value	OR	95% CI	*p*-Value
Cancer																		
No	—	—		—	—		—	—		—	—		—	—		—	—	
Yes	3.23	2.77, 3.77	<0.001	1.41	1.18, 1.67	<0.001	1.33	1.11, 1.59	0.002	1.12	0.93, 1.34	0.2	1.12	0.93, 1.36	0.2	1.16	0.95, 1.41	0.2

We applied weighted logistic regression models to estimate the odds ratios (ORs), 95% CI, and *p* value for heart failure associated with cancer status, alongside other demographic and clinical variables. Three models were assessed both pre- and post-PSM: Model 1 was unadjusted; Model 2 adjusted for gender, age, race, education, poverty income ratio, and marital status; Model 3 included additional adjustments for marital status, diabetes, blood pressure, smoking, body mass index (BMI), and waist circumference.

**Table 4:** Weighted logistic regression results for HF status on cancer.

	Model 1 Pre-PSM			Model 2 Pre-PSM			Model 3 Pre-PSM			Model 1 Post-PSM			Model 2 Post-PSM			Model 3 Post-PSM		
Characteristic	OR	95% CI	*p*-Value	OR	95% CI	*p*-Value	OR	95% CI	*p*-Value	OR	95% CI	*p*-Value	OR	95% CI	*p*-Value	OR	95% CI	*p*-Value
Heart Failure																		
No	—	—		—	—		—	—		—	—		—	—		—	—	
Yes	3.23	2.77, 3.77	<0.001	1.39	1.18, 1.64	<0.001	1.31	1.11, 1.55	0.002	1.41	1.14, 1.74	0.002	1.48	1.19, 1.84	<0.001	1.46	1.17, 1.82	<0.001

This study conducted weighted logistic regression to evaluate the relationship between heart failure and cancer occurrence. Both pre- and post-propensity score matching (PSM) were applied to three models. Model 1 provided unadjusted odds ratios (ORs). Model 2 adjusted for gender, age, race, education, poverty income ratio (PIR), and marital status. Model 3 further adjusted for diabetes, smoking, moderate activity, and hepatitis B and C status.

## Data Availability

The raw data supporting the conclusions of this article will be made available by the authors, without undue reservation.
